# Taking the pain out of pain

**DOI:** 10.1177/20494637231208167

**Published:** 2023-10-18

**Authors:** Catharine Montgomery, Helen M Poole, Emma Begley, Yasir Abbasi

**Affiliations:** 1 School of Psychology, 4589 Liverpool John Moores University, Liverpool, UK; 2 School of Psychology, 1722 Aston University, Birmingham, UK; 3 Via, London, UK

**Keywords:** Chronic pain, pain, pain clinics, pain management, pain measurement

## Dopesick of painkillers?

While the potential risks of opioid prescribing for chronic non-cancer pain (CNCP) have long been known to addiction psychiatrists and pain researchers and clinicians, recent popular dramatizations of the opioid epidemic in the USA have brought the issue in to mainstream public sight. Hulu’s 2021 production *Dopesick* and Netflix’s 2023 release *Painkiller* both tell the story of the manufacturer of Oxycontin (Oxycodone), Purdue Pharma, and how development, testing and false and aggressive marketing of Oxycontin led to the current USA opioid crisis. Currently opioid consumption in the USA is almost double of any other country, consuming on average over 40,000 tons (per million inhabitants) they continue to rank the highest across the globe (INCB, 2019), compared to the UK who rank 15th. Moreover, the USA was identified as the world’s largest consumer of opioids with a 400% increase between the period 2000 and 2010, compared with a 65% increase in the UK during that time (Häuser et al., 2018). It is therefore not surprising that the USA has received a lot of attention in recent media and have declared a state of crisis regarding the prescription of opioid drugs for the treatment of CNCP.

UK based prevalence studies demonstrate historical increases in opioid prescribing (Begley et al., 2023; Chen et al., 2019; Foy et al., 2016; Mordecai et al., 2018; Torrance et al., 2018; Zin et al., 2014). A recent report found that during 2018 there were 5.6 million adults living in England receiving at least one opioid, and despite the historical increasing trend, a decrease in prescribing, compared with 2017 (Taylor et al., 2019). Codeine and Morphine are the most commonly prescribed weak and strong opioids respectively (Begley et al., 2023; BMA, 2017; Torrance et al., 2018), with oxycodone having the greatest increase in annual number of prescriptions in one analysis (Zin et al., 2014). Zin et al. (2014) specifically investigated UK prescribing trends between 2000 and 2010, finding that morphine was the most frequently prescribed opioid and contributor to higher daily doses (above 50 mg Morphine Equivalent Dose - MED). Oxycodone, buprenorphine, and fentanyl also increased over time, however oxycodone and to a lesser extent fentanyl were specifically linked to doses exceeding 200 mg MED/day. Conversely, despite the number of strong opioid prescriptions doubling in the UK, over 50% of people reporting severe pain in Scotland were not prescribed an opioid analgesic (Torrance et al., 2018).

## Why is opioid reliance a problem?

Opioid use for CNCP has been linked to increased risk of heart attack, fractures, overdose, and death (Chou et al., 2015). There is growing evidence that the risk of side-effects and adverse events is linked to medium and long-term opioid use (Els et al., 2017) and is dose dependent (Bedson et al., 2019; Chou et al., 2015; Dunn et al., 2010). In one US study with 9940 CNCP patients, risk of overdose was 8.9 times more likely in those taking daily doses above 100 mg MED (Dunn et al., 2010). Furthermore, a systematic review investigating the effectiveness and risk of long-term opioid therapy for CNCP, reported that risk of abuse and dependence increases in doses above 120 mg MED, and found guidance implementing a 120 mg MED threshold decreased this risk of harm (Chou et al., 2015). It is concerning therefore, that in the UK there is clear indication that opioids are being prescribed long-term, are likely to increase in dose and strength and coincide with CNCP complaints (Foy et al., 2016; Zin et al., 2014). The most common side effects of using opioids include gastrointestinal problems (e.g., constipation, nausea or vomiting), tolerance (inducing hyperalgesia), dizziness, fatigue, hot flushes, itching, and sexual dysfunction (Els et al., 2017; Grover et al., 2014; Harris, 2008). Some of these effects are likely to improve with continued use, though constipation and tolerance are unlikely to diminish (FPM, 2015). On the other hand, the incidence of serious adverse events (e.g., dependence, overdose, fractures, heart attack or death) have been correlated to long-term, dose dependent use (Bedson et al., 2019; Chou et al., 2015; Dunn et al., 2010). There is also some evidence to suggest that opioids do not improve or even worsen functionality and levels of pain in CNCP patients (Häuser et al., 2015). For example, a Danish national health survey that followed 2354 CNCP participants, out of 10,434 who completed the survey 5 years previously, found that rates of recovery were four times higher among those who didn’t use opioids (Sjøgren et al., 2010). Comparing two surveys (in 2000 and 2005) Sjøgren found that age, level of education, obesity, self-reported quality of life and physical job strain predicted chronic pain, so perhaps improvements in these variables should be considered in promoting pain recovery.

Moreover, the US have linked the growing levels of drug related deaths and overdoses to the increase of opioid prescribing, and common prescribing practices involving high-dose long-term prescriptions (Phillips et al., 2017). These conclusions come largely from several studies investigating opioid prescription overdose deaths and dispensing data (Boudreau et al., 2009; Paulozzi et al., 2011; Von Korff et al., 2008). For example, Paulozzi et al., (2011) identified that 73.8% of overdose deaths were attributed to opioid pain prescriptions which also coincided with an increase in sale data during 1999–2008 (Paulozzi et al., 2011). It is difficult to accurately measure the number of deaths attributed to prescription opioids in the UK, as information on death certificates only identifies the presence of drugs at time of death, and not whether death was caused by a specific substance (FPM, 2016). Links may be drawn using data from the Office of National Statistics (ONS), which indicate that over half of all drug related deaths since 2006 have involved an opioid (namely heroin or morphine). Specifically in 2022 there were 2219 deaths (45.7% of drug-related poisonings) where an opioid was mentioned in the death certificate (ONS, 2023).

## What can we do?

Leading healthcare authorities in the UK agree that there are no likely benefits of long-term high dose opioid prescribing and that patients’ risk of harm increases in doses above 120 mg MED (FPM, 2015). As a result, an online resource was developed in 2015 (Opioids Aware) to provide both clinicians and CNCP patients with advice about using opioids for CNCP. The resource advises when Health Care Professional’s (HCPs) should consider weaning or discontinuing opioids (e.g., when risks outweigh the benefits), and factors to consider when preparing to wean patients (e.g., providing an explanation, agreeing outcomes, monitoring). Although this is useful, there remains no formal guidelines on how to safely manage withdrawal or continuing symptoms of pain among this population, though there are a number of feasibility studies which are at varying stages of reporting (e.g. Poole et al., 2023; Sandhu et al., 2023). In 2020, growing concern of the increased risk of harm with using opioids to treat CNCP long-term provoked a shift in national guidance. NICE now advise against the use of opioids in the treatment and management of CNCP and instead advocate the use of non-pharmacological therapies (NICE, 2020). For patients already on historical opioid prescriptions however, there is a lack of specialist services offering opioid tapering with psychological support, meaning high dose long-term opioid patients (“legacy patients”), may face potentially life-threatening harms if not managed appropriately (Darnall et al., 2019). Moreover, while opioid reduction has demonstrated long-term benefits, increases in pain and opioid withdrawal syndrome during tapering can have subsequent consequences for patient safety and public health as detailed below.

## What are the potential problems?

While many patients can taper successfully with clinician support, for some patients the physiological and psychological effects of opioids, and the fear of the unknown (if not opioids then what?), may result in the sourcing and self-directed use of prescription and non-prescription opioids. There has been an increased interest in online sourcing of opioids (particularly synthetic high strength opioids such as fentanyl; Hadland & Beletsky, 2018) with the European Monitoring Centre for Drugs and Drug Addiction (EMCDDA, 2016) developing an early warning system for surveillance of trends in their use. A recent analysis using online search data found that Oxycodone and Morphine were the two most searched prescription medications, and that this increased in 2020, presumably while the public had more limited access to clinicians during the COVID-19 restrictions (Whitfield et al., 2021). While there is greater regulation over online pharmacies and prescription diversion in the UK compared to the USA (General Pharmaceutical Council, 2019), there is still need for increased pharmacovigilance. For example, the tightening of prescribing guidelines in the USA was not followed by a comparable reduction in opioid-related mortality (Kerr, 2019), potentially suggesting that opioid sourcing has moved to illicit substitutes (Whitfield et al., 2021). The increase in internet sourcing of opioids is also concerning due to the increase in high-potency synthetic opioids that are available in online marketplaces. Recent analyses of illicit opioid samples have demonstrated the presence of high-potency synthetic opioids such as, nitazenes which have higher potency and toxicity than fentanyl (Pergolizzi et al., 2023). We should be mindful however, not to perpetuate widespread panic of a UK based opioid epidemic. As Els and co-workers argue, panic emerges from an accumulation of misperceptions, lack of alternative treatment and over reliance on low quality studies (Els et al., 2017) in this area. More specifically, Sullivan and Howe (2013) postulate that the actions of physicians (e.g. lack of knowledge or access to alternative treatment), patients (e.g. priority of pain relief instead of improved function) and society (e.g. attitudes toward opioid therapy for CNCP) would facilitate such a crisis (Sullivan & Howe, 2013). For acute pain, opioids remain an effective analgesic and do work well for some patients and some conditions. Rieder (2010) discussed the ethical and moral responsibility associated with opioid prescribing, concluding that clinicians should not be anti-opioid, as the alternative option risks creating another crisis, a pain crisis (Rieder, 2010).

## How do we take the pain out of pain?

It is important to note that the majority of patients prescribed opioids to treat their pain do not misuse them (Fishbain et al., 2008; Higgins et al., 2018; Vowles et al., 2015), though the pain relieving qualities and increased tolerance to opioids may lead to over reliance (Phillips et al., 2017). The UN highlights a number of key trends that reflect the global picture, and despite an overall downward trend in the global opium movement (i.e. production, stock and use) since 2005, slight increases are evident during 2011 and 2017. This downward trend perhaps indicates a decrease of demand being placed on opioids but equally could reflect global responses to control prescribing and misuse of legally acquired opioids. Globally, 2017 marked the lowest level of opium importation in 20 years, though consumption of morphine has still roughly doubled since 1997 (INCB, 2019). While the UK was on an increasing trajectory of opioid prescribing until 2018 (e.g. Jani et al., 2020), new analysis from NHS England shows that opioid prescriptions have fallen by 8% in the last 3 years (Ashworth et al 2023), perhaps indicating a shift from opioid reliance, to self-management of pain in conjunction with increased prescribing of other medications, such as, gabapentinoids (Ashworth et al., 2023). This will be welcome news for some patients, as accounts of patients’ lived experience of being treated with opioids, demonstrate that they perceive opioids to be both a salvation and a curse (Ljungvall et al., 2020). Ljungvall et al. (2020) highlight that patients don’t particularly like taking opioids, and that the stigma attached to them make them feel that they are a nuisance or drug seeking when they discuss with their clinician that treatment is not working.

For other patients however, particularly those with complex needs and co-existing conditions, a truly holistic multidisciplinary support service will be needed. Such a service must include data scientists for early identification of those who are at potential risk (e.g. long-term, escalating dose, high dose, high potency opioids), and issue timely, patient-centric referrals to services with addiction services, primary care, hospitals and pain clinics all providing support. Only then can we really start taking the pain out of pain.
